# Regulation of Microglial Functions by Purinergic Mechanisms in the Healthy and Diseased CNS

**DOI:** 10.3390/cells9051108

**Published:** 2020-04-29

**Authors:** Peter Illes, Patrizia Rubini, Henning Ulrich, Yafei Zhao, Yong Tang

**Affiliations:** 1 Rudolf Boehm Institute for Pharmacology and Toxicology, University of Leipzig, 04107 Leipzig, Germany; 2 International Collaborative Centre on Big Science Plan for Purine Signalling, Chengdu University of Traditional Chinese Medicine, Chengdu 610075, China; patrizia.rubini@cdutcm.edu.cn (P.R.); tangyong@cdutcm.edu.cn (Y.T.); 3 Department of Biochemistry, Institute of Chemistry, University of São Paulo, São Paulo 748, Brazil; henning@iq.usp.br; 4 Acupuncture and Tuina School, Chengdu University of Traditional Chinese Medicine, Chengdu 610075, China; zhaoyafei@stu.cdutcm.edu.cn

**Keywords:** surveying microglia, amoeboid microglia, P2X receptors, P2Y receptors, P1 receptors, CD39, CD73, microglia-neuron crosstalk, phagocytosis, microglial products, neuroinflammation

## Abstract

Microglial cells, the resident macrophages of the central nervous system (CNS), exist in a process-bearing, ramified/surveying phenotype under resting conditions. Upon activation by cell-damaging factors, they get transformed into an amoeboid phenotype releasing various cell products including pro-inflammatory cytokines, chemokines, proteases, reactive oxygen/nitrogen species, and the excytotoxic ATP and glutamate. In addition, they engulf pathogenic bacteria or cell debris and phagocytose them. However, already resting/surveying microglia have a number of important physiological functions in the CNS; for example, they shield small disruptions of the blood–brain barrier by their processes, dynamically interact with synaptic structures, and clear surplus synapses during development. In neurodegenerative illnesses, they aggravate the original disease by a microglia-based compulsory neuroinflammatory reaction. Therefore, the blockade of this reaction improves the outcome of Alzheimer’s Disease, Parkinson’s Disease, multiple sclerosis, amyotrophic lateral sclerosis, etc. The function of microglia is regulated by a whole array of purinergic receptors classified as P2Y12, P2Y6, P2Y4, P2X4, P2X7, A2A, and A3, as targets of endogenous ATP, ADP, or adenosine. ATP is sequentially degraded by the ecto-nucleotidases and 5′-nucleotidase enzymes to the almost inactive inosine as an end product. The appropriate selective agonists/antagonists for purinergic receptors as well as the respective enzyme inhibitors may profoundly interfere with microglial functions and reconstitute the homeostasis of the CNS disturbed by neuroinflammation.

## 1. Introduction

The human central nervous system (CNS) consists of neuronal and non-neuronal cells in approximately a 1:1 relationship [1,2]. Glia constitute most of the non-neuronal cell population, but other cell types such as pericytes and endothelial cells are also part of it. On the basis of morphological criteria, in the human neocortex, oligodendrocytes (including oligodendrocyte precursor cells, NG2 glia) account for about 50–75% of the total glial population, astrocytes for 20–40%, and microglia for 5–10% [3,4]. Microglial cells are resident macrophages and the most important effectors of the brain’s innate immunity [5,6]. The origin of microglia has been the subject of a long-standing debate, which reached an end a couple of years ago with the recognition that in spite of their similarity to peripheral macrophages, these cells are of different genetic origin. Macrophages are continuously produced in the bone marrow during the post-natal stage, whereas microglia are derived from yolk-sac progenitors migrating into the CNS by starting at embryonic day 8.5 and continuing this migration until the blood–brain barrier is formed [7]. Lineage-specific genes (e.g., *Pu.1, Irf8*) define the microglial transcriptional network and distinguish it from that of tissue-resident macrophages [8].

Microglia exist in a highly ramified form under resting conditions but get activated by changes in brain homeostasis [9]. This leads eventually to polarization into an amoeboid form which is able to phagocytose pathogenic bacteria and releases a number of bioactive molecules such as pro-inflammatory cytokines (interleukin-1β (IL-1β), tumor necrosis factor (TNF-α), chemokines, proteases, reactive oxygen/nitrogen species, and probably also the excitotoxic ATP and glutamate by vesicular exocytosis) [10]. In addition to this classically activated M1 microglial/macrophage phenotype, typically releasing the mentioned destructive pro-inflammatory mediators, the alternatively activated M2-phenotype clears cellular debris through phagocytosis and releases numerous protective factors (IL-4, IL-13, nerve growth factor (NGF), fibroblast growth factor (FGF)) [11]. Whereas M1 microglia are supposed to participate in immunological defense mechanisms of the CNS, M2 microglia are involved in regenerative mechanisms accompanying long-lasting neurodegeneration in, e.g., Alzheimer’s Disease (AD) or Parkinson’s Disease (PD). In addition to the differential release of inflammatory and protective factors from M1 and M2 microglia, respectively, the two phenotypes are characterized by different cell surface markers (e.g., M1: CD11b, CD16; M2: CD163, CD206) [12,13]. Further, in vitro treatment of microglia with lipopolysaccharide (LPS) induces transformation to M1 microglia, whereas treatment with the autocrine cytokine IL-4 induces transformation to M2 microglia [14].

However, recent evidence indicates that the M1/M2 dichotomy is an oversimplified conceptual framework that only represents two extreme activation states. Firstly, M2 microglia/macrophages are divided into three major types based on their roles (M2a, M2b, M2c) [15,16], and secondly, a mixture of M1/M2 phenotypes have been also reported [17]. It is now clear that there is not either a single nor a discrete number of microglial reactive states but a diversity of phenotypes that are determined by a fine detection of environmental cues, which allows microglial cells to perform specific functions in different physiological and pathological conditions [16]. In spite of these limitations, the M1/M2 concept is generally accepted as a working model used to describe microglial phenotypic appearances.

After having conveyed some useful general knowledge on microglial cells, we will discuss the physiological/pathophysiological functions of microglia.

## 2. Physiological Roles of Microglia

### 2.1. Comparability of Results Obtained on Microglia Under In Vivo and In Vitro Conditions

Mechanical damage inflicted upon brain tissue during the preparation of primary cell cultures activates resting microglia; therefore, results from cell cultures should be interpreted with caution (microglia has morphological and functional properties different from the in vivo situation); results obtained from the superficial layers of brain slice preparations may also supply properties only relevant for activated microglia (in deeper layers, the conditions are more comparable to the in vivo situation) [16]. Because we deal in this review article with purinergic receptors of microglia and the (patho)physiological effects mediated by them, it is important to find out whether these effects are comparable in cultured preparations and synaptically wired in vitro experimental systems.

**Figure 1:**
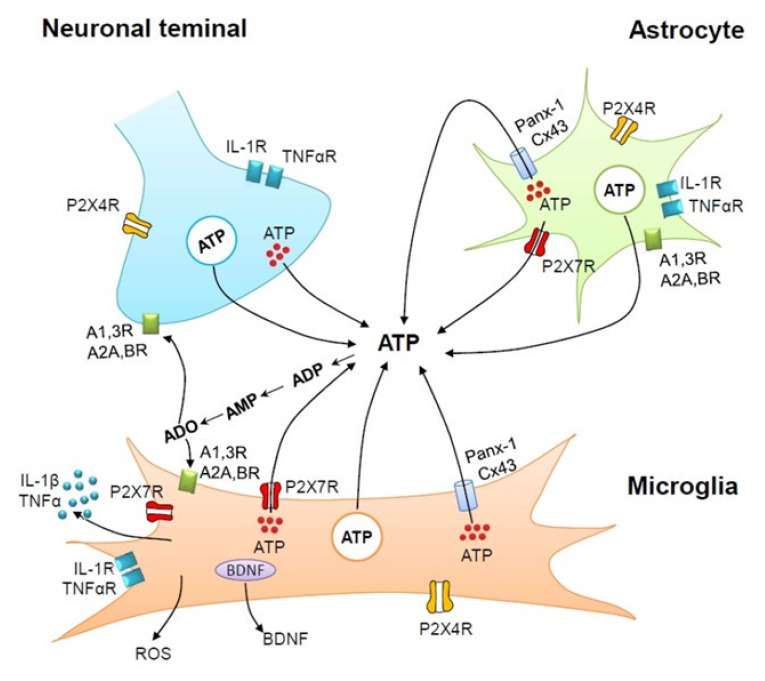
Purinergic receptors as well as ATP release mechanisms participating in neuron-astrocyte-microglia cross-talk. Microglia possess the ligand-gated P2X4 and P2X7 receptor subtypes as well as all subtypes of the G protein-coupled P1 (A1, A2A, A2B, A3) receptors. In addition, microglia release ATP from synaptic/lysosomal vesicles via exocytosis but also via connexin (mainly Cx43) channels and pannexin (Panx-1) hemichannels. P2X4 receptor (R) activation induces the vesicular release of brain-derived neurotrophic factor (BDNF), which causes neuropathic pain in the dorsal horn spinal cord. P2X7R activation results in the outward blebbing of the microglial plasma membrane and the production of extracellular vesicles containing interleukin-1β (IL-1β). The pro-inflammatory cytokines IL-1β and tumor necrosis factor-α (TNFα) bind to their receptors IL-1R and TNFαR, respectively. P2X7R activation induces the diffusion of reactive oxygen species (ROS) through the plasma membrane. All these microglial products cause neuroinflammation and neurodegeneration. Exocytotic, Ca^2+^-dependent, vesicular release occurs from neurons, astrocytes, and microglia. The vesicular release of ATP from neurons is much faster than that from astrocytes or microglia, although the vesicular proteins involved in exocytosis are relatively similar in the three cell types. ATP is rapidly degraded by ecto-nucleotidases to ADP, AMP, and eventually by 5’-nucleotidase to the bioactive adenosine (ADO). Artwork by Dr. Haiyan Yin.

Microglia respond to neurotransmitter release and neuronal activity at nearby synapses, although, in contrast to isolated/cultured microglia [18], these cells, when located in brain slices or in the intact brain, are largely devoid of receptors for most neurotransmitters [19]. However, they exhibit a plethora of P2X and P2YR subtypes which may be activated by ATP released from neurons following the stimulation of their receptors by other neurotransmitters [2]. Purinergic receptors, as well as ATP release mechanisms participating in neuron–astrocyte–microglia cross-talk, are illustrated in Figure 1 (see also Section 5).

The group of Helmut Kettenmann noticed as early as 1993 that cultured microglial cells prepared from the mouse brain respond to ATP at the resting membrane potential with an early inward cationic current and a late outward K^+^ current [20,21]. The cultured cells were slightly elongated but process-free, thereby exhibiting morphological characteristics of amoeboid microglia [20]. The non-selective cationic current was considered to be due to the activation of P2X, and the late K^+^ current to the activation of P2Y receptors (Rs). These effects of ATP could be reproduced in microglial cells of acute brain slices identified by staining with Texas Red-coupled tomato lectin [22,23]. In mouse corpus callosum slices, the cells recorded from showed the typical morphology of resting, process-bearing microglia [23].

Somewhat later, a similar situation was observed to occur in rat cortical astrocytes. both in cell culture and in brain slices. The highly plastic astrocytes exhibited, instead of their typical process-bearing shape, a confluent, background layer of flat cells on the base of the culture dish [24], while in brain slices prepared from the rat prefrontal cortex, fluorescence microscopy of Lucifer Yellow-filled astrocytes showed cells surrounded by dense processes extending several micrometers around the somata [25]. Nonetheless, the P2X7R currents recorded from microglia in primary cultures and brain slices could not be discriminated from each other either by physiological (reversal potential measurement) nor pharmacological (selective agonists and antagonists) means.

Based on these and similar observations, we tentatively suggest that whereas cultured microglia/astrocytes may only incompletely model the in vivo situation for a variety of CNS signalling molecules, ATP/ADP and even adenosine effects are much more stable at different levels of synaptic organization or their absence (primary cell cultures, acutely isolated cells, brain slice preparations, intact brain). The reason for this phenomenon may be that ATP/ADP/adenosine are primitive signalling molecules appearing very early in phylo- and ontogenesis and being quite enduring in their functional properties [26,27]. Therefore, we decided to indicate only in a few cases when tissue cultures were used for investigations.

### 2.2. Microglial Process Motility

In order to investigate microglial cells in their natural environment, transgenic mice (CX3CR1-EGFP; [28]) were used which showed specific expression of enhanced green fluorescent protein (EGFP) in resident microglia of the CNS. CX3CR1 (R stands for receptor) is a chemokine receptor highly specific to microglia; the chemokine fractalkine (CX3CL1; L stands for ligand) is present in neurons and signals to microglia via this receptor [29]. Time-lapse imaging experiments were acquired transcranially by using thinned-skull preparations in vivo. The somata of microglial cells remained fixed for rather long periods of time, while the microglial processes were remarkably motile. These processes rapidly approached a small laser ablation caused by the two-photon laser [30] or disruption of the blood–brain barrier and provoked a shielding of the injured site [31]. It was most interesting to notice that the release of ATP via astrocytic hemichannels may be a chemoattractant to induce site-directed movement of microglial processes [30]. It was concluded that resting microglia continuously survey the parenchymal environment within non-overlapping territories with a multitude of fine, exceptionally motile processes/protrusions and sense tissue abnormalities [32,33].

### 2.3. Microglia–Neuron Crosstalk at Synaptic Structures

Microglia are well-positioned to monitor neuronal firing and synaptic function; in response to neuronal activity, microglia steer their processes towards active synapses [34,35]. In adult mouse visual cortex, dynamic interactions between highly motile microglial processes and synaptic structures were observed under non-pathological conditions by two-photon imaging [34]. In this study, microglial processes were found to interact with axon terminals and dendritic spines in a transient manner. Reducing neural activity by enucleating both eyes induced retraction of microglial processes.

The ‘tripartite synapse’ hypothesis suggests that presynaptic neuronal elements, postsynaptic dendritic specializations, and astrocytic processes that contact or even enwrap the synapse, together form a mutually interacting unit influenced by neuro- and glio-transmitters [36]. The realization that microglial processes also contact synaptic structures and thereby establish a dynamic crosstalk between astrocytes, microglia, and neurons broadened this concept to the idea of a ‘quadpartite synapse’ [37]. It has been shown that neuronal ATP may modulate the microglial secretion of certain cytokines [38]. In return, microglia influence and modulate neuronal functions by the release of cytokines, prostaglandins, and neurotrophic factors [37]. In accordance with this assumption, microglia could regulate the basal glutamatergic and GABAergic synaptic transmission [39].

### 2.4. Microglial Phagocytosis

**Figure 2:**
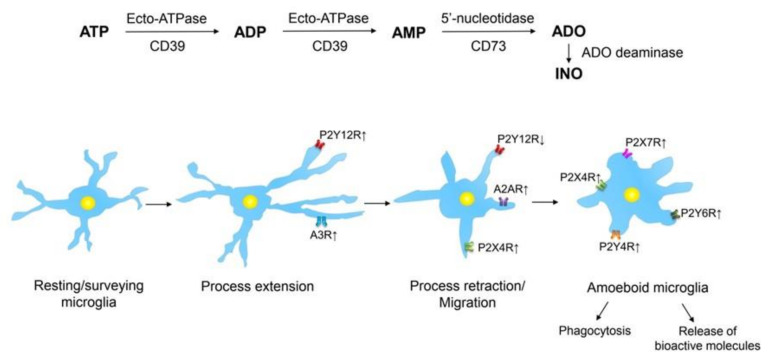
Purinergic receptors at microglial cells exemplifying their different activation states. ATP is sequentially dephosphorylated by an enzymatic cascade to AMP by ecto-ATPase (NPDase-1; CD39) through the intermediary product ADP. AMP is further degraded to adenosine (ADO) by 5’-nucleotidase (CD73). Finally, adenosine is almost inactivated by adenosine deaminase to inosine (INO). Resting/ramified microglia extend and retrieve processes, thereby scanning their territories, non-overlapping with those of the neighboring microglial cells. When ATP is released/outpoured into the extracellular space from damaged CNS cells, as a first step of microglial activation, these cells extend their processes towards the site of injury triggered by stimulation of P2Y12 receptors (Rs). Both P2Y12 and A3Rs are upregulated in consequence of CNS damage, and they co-operate in steering the microglial process extension. Subsequently, these processes retract due to the downregulation of P2Y12Rs and the upregulation A2ARs; the migratory activity of this microglia is controlled by the interaction of P2Y12 and P2X4Rs. After the complete retraction of the microglial processes, an amoeboid phenotype is evolving. On this microglia, phagocytosis and pinocytosis are induced by P2Y6R and P2Y4R activation, respectively. P2X4Rs mediate the secretion of brain-derived neurotrophic factor (BDNF) in spinal cord microglia. P2X7Rs may initiate multiple secretory processes such as the release of pro-inflammatory cytokines, chemokines, growth factors, proteases, reactive oxygen/nitrogen species, cannabinoids, and probably also the excitotoxic ATP and glutamate. Upwardly directed arrows beside receptors indicate their upregulation or increased activation by agonists, while downwardly directed arrows indicate their downregulation or decreased activation by agonists. For further details, see [19,79,80]. Artwork by Dr. Haiyan Yin.

It has been known for a long time that neuronal damage leads to the activation of ramified microglia through several steps transforming them by multiple factors, such as colony-stimulating factor, LPS, and interferon-γ, into the amoeboid morphology [40,41]. More recently, it became generally accepted that macrophage/microglia activation is not an all-or-none process; it may be partially reversible and depends on the pathological context, the nature and strength of the stimuli, and the settings in which these stimuli appear [16,42]. With these limitations in mind, the reader should forgive us for illustrating microglial activation because of didactic reasons in Figure 2 as a linear process.

Complement receptors appear to steer microglial phagocytosis. It was shown that C1q, the initiating protein in the classical complement cascade, is expressed by postnatal neurons and is localized to synapses throughout the CNS [43]. Mice deficient in complement protein C1q or the downstream complement protein C3 exhibit large sustained defects in CNS synapse elimination, leading to abnormal neuronal function. In conclusion, it is hypothesized that unwanted synapses are tagged by complement and are subsequently eliminated by microglial activity induced by the binding of this complement to its microglial pattern recognition receptor CR3.

In fact, microglia engulf presynaptic inputs in the reticulogeniculate system of the visual pathway, and engulfment is dependent upon neuronal activity as well as the microglia-specific phagocytic signal transduction [44]. This was shown by the use of mice lacking functional CR3 or mice deficient in the CR3 ligand C3; the knockout animals did not show the microglial reactions aimed at eliminating synapses. Hence, it was concluded that based on the perception of synaptic activity, microglia are able to remove excess synapses by synaptic pruning and thereby shape postnatal neuronal circuits [44].

Further evidence for microglial phagocytosis was obtained from investigations of the fractalkine (CX3CL1) pathway (see Section 2.2). Young CX3CR1 knockout mice show a significant reduction in the density of microglia during the postnatal period and exhibit transient defects in synaptic connectivity and plasticity in the postnatal hippocampus [45]. These mice reveal an increase in dendritic spine density and postsynaptic density protein 25 (PSD-95) immunoreactivity, enhanced hippocampal long-term depression, and decreased duration of latency to pentylenetetrazol-induced seizure responses, characteristically associated with less mature synapses. It was considered that synaptic pruning by microglia is targeted to both pre- and postsynaptic elements and is necessary for normal brain development which is absent in these knockout animals [45].

In contrast to the idea of microglial ‘phagocytosis’ of entire synapses, it was reported that only ‘trogocytosis’ (synaptic nibbling) occurs [46]. This means elimination of presynaptic boutons and axons, with no evidence for elimination of postsynaptic material. Intriguingly, microglia contacts at postsynaptic sites frequently elicited transient filopodia, most of which originated from mature spines. It is quite possible that the neuronal damage is preferentially presynaptic [44], with some denervation-type secondary changes on the postsynaptic side [45].

In addition to synaptic pruning, microglia may sculpt neuronal circuits during development by selective phagocytosis of neural stem cells (NSCs) [47,48]. Neurons of the adult cerebral cortex are generated in the ventricular and subventricular zones during prenatal development [49]. Phagocytosis of overtly produced cells by microglia is essential, especially at the end stages of cortical neurogenesis [48]. Unrestrained cell production during prenatal brain development would have profoundly negative consequences for brain organization and function. Not only surplus NSCs, but freshly generated redundant neurons may also be killed and eliminated by microglial apoptosis and subsequent phagocytosis [50,51].

## 3. Pathological Roles of Microglia-activation Processes

As previously mentioned, microglia get activated in response to changes in brain homeostasis. These cells acquire phagocytic properties and the ability to release a number of pro-inflammatory molecules. The ATP-sensitive P2X7R, which is a subtype of the ligand-gated P2XR family (see Section 4.1 and Section 4.2.4), is a major driver of inflammation [52]. This receptor is stimulated by large concentrations of ATP outpouring from CNS cells under noxious conditions during both acute injury (e.g., trauma, hypoxia/ischemia or epilepsy-induced seizures) and chronic neurodegenerative illnesses (e.g., AD, PD, amyotrophic lateral sclerosis, and multiple sclerosis) [53].

In the CNS, P2X7Rs are preferentially localized on microglial cells [54]. Microglia are equipped with a battery of pattern recognition receptors that stereotypically recognize pathogen-associated molecules (PAMPs) that warn of the presence of exogenous material such as components of the bacterial cell wall (e.g., lipopolysaccharide (LPS) acting on toll-like receptor TLR4) or repeats of bacterial or viral nucleic acids [55]. Microglia also possess a wide range of surface molecules sensing danger-associated molecular patterns (DAMPs), such as scavenger [56], purine P2X7 [57], and cytokine/chemokine receptors; the stimulation by PAMPs/DAMPs results in the activation of resting microglia [58].

The activation process can be traced by bulk RNA [59] and single-cell RNA [60] sequencing, as well as by epigenetic [61] and proteomic analyses [16,62]. These investigations lend further support to the most heterogeneous nature of this process in microglial cells. Based on single-cell RNA sequencing data, homeostatic microglia adopt distinct disease-associated microglia (DAM) profiles in amyloid β (Aβ) protein plaques of AD brains [60,63]. Aβ is one of the neurodegeneration-associated molecular patterns (NAMPs) that are commonly present in various pathogenous CNS conditions and are recognized by receptors constitutively expressed on microglia triggering their transition/activation into DAM [64]. In conclusion, DAM microglia have been identified as a subpopulation of microglial cells in experimental models of neurodegenerative illnesses such as AD, frontotemporal dementia, and amyotrophic lateral sclerosis [64].

## 4. P1 and P2 Purinergic Receptors Involved in Microglial Functions

### 4.1. Receptors and Inactivation Mechanisms for ATP/ADP and Adenosine

ATP not only supports energy storage within cells but is also a transmitter/signalling molecule that serves intercellular communication [65]. Receptors for ATP have been classified into two types, the ligand-gated cationic channel P2X (seven mammalian subtypes: P2X1, 2, 3, 4, 5, 6, and 7) and the G protein-coupled P2YRs (eight mammalian subtypes: P2Y1, 2, 4, 6, 11, 12, 13, and 14) [66]. P2XRs consist of assemblies of three identical or divergent subunits, thereby forming homo- or heteromeric channels [67]. The P2X7R is unique in occurring only as a homotrimer and causing, in addition to immediate effects, molecular changes on a much longer time scale, such as proliferation and apoptosis [68]. Numerous detailed review articles discuss evidence for the presence of P2X/P2YRs on microglia and the functional consequences of their activation (e.g., [69,70,71]); this information will be updated in the present overview.

P2R signalling is terminated by the conversion of ATP/ADP to adenosine within the extracellular compartment by the activity of ecto-nucleotidases. The four main groups of ectonucleotidases are the ecto-nucleoside triphosphate diphosphohydrolases (NTPDases), ecto-5′-nucleotidase, ectonucleotide pyrophosphatase/phosphodiesterases, and alkaline phosphatases [72,73]. The degradation of ATP/ADP to adenosine generates an agonist with multiple, sometimes opposite, effects to those of its mother molecules. Adenosine may act at four types of receptors, termed A1, A2A, A2B, and A3 [74]. All adenosine receptors are G protein-coupled; A1/A3Rs inhibit adenylate cyclase activity via coupling to Gi/o, while A2A/A2BRs stimulate this enzyme via G_s/olf_. The effect of adenosine is terminated by enzymatic degradation to the only slightly active inosine by adenosine deaminase [75] or by enzymatic conversion to AMP by adenosine kinase [76]. However, extracellular adenosine is also rapidly taken up into the surrounding tissue by the equilibrative nucleoside transporter isoenzymes ENT1-4 [77].

All these receptors and enzyme systems form a complex network sometimes called the ‘purinome’ [78]. Within this network, the enzymatic pathways generate agonists for a wide range of receptors, which results in a huge diversity of sometimes even contradictory responses. In the present review article, we will concentrate on those receptors which are important for the regulation of microglial functions (Figure 2). It has to be mentioned that depending on the activation state of microglia, the densities as well as the agonist sensitivities of their purinergic receptors become modified [19,79,80].

### 4.2. Regulation of Physiological and Pathological Functions of Microglia by ATP/ADP, UTP/UDP, and Adenosine; Chemotaxis and Secretory Properties

#### 4.2.1. P2Y12 and P2Y13 Receptors

As mentioned earlier, resting/surveying microglia continuously scan a given territory of their parenchymal environment with a multitude of fine and exceptionally motile processes/protrusions and record tissue abnormalities (see Section 2.1). While one of the original reports on this phenomenon already delivered convincing proofs for the participation of ATP/ADP in the motility of the microglial processes [30], a more recent publication characterized the receptor involved as belonging to the ADP-sensitive P2Y12-type [81]. The P2Y12R was initially identified on blood platelets, where it regulates their conversion from the inactive to the active state during the blotting process [82]. The molecular changes triggered by P2Y12R activation appear to be similar in platelets and microglia, but of course with different functional consequences. Protruding microglial processes may shield minor sites of endothelial/astrocytic/neuronal damage in the CNS [31]. More massive disturbances of CNS homeostasis, however, result in strong activation of microglia, adapting an amoeboid morphology; this causes migration of the microglial cell body towards the source of released ATP [83].

We discussed already that microglial processes contact synaptic structures and thereby establish a dynamic cross-talk with neurons [37]. However, a recent publication raised an alternative, most interesting hypothesis [84]. The authors of this study identified the site of interaction between microglial processes and cortical layer 2/3 neurons at the cell body rather than at synaptic elements, including axonal boutons and dendritic spines, in the adult brain of CRXCR1-GFP transgenic mouse. Metabolic activity of neuronal mitochondria was linked by the production of ATP and its somatic, vesicular release, with the rapid formation of microglial junctions. In consequence, the protrusion of microglial processes was an immediate reaction to neuronal activation and was blocked by inhibition of P2Y12Rs.

Microglial process extension, and as a non-obligatory second step, the transition of resting to amoeboid microglia and its migratory movement, is due to the P2Y12R-induced activation of phosphatidylinositol 3′-kinase (PI3K) and phospholipase C (PLC) signalling pathways [85]. It was reported that the PLC-mediated increase in intracellular Ca^2+^ concentration-triggered proteinkinase B (Akt) activation was also involved in the second-messenger mechanisms of P2Y12Rs [85]. Eventually, P2Y12Rs initiate an adhesive interaction with the surrounding extracellular matrix through integrins [86].

Nerve-injury-activated microglia engulf myelinated axons in the spinal cord dorsal horn in a P2Y12R-triggered and p38 mitogen-activated protein kinase signalling dependent manner; this reaction is causally involved in the pathogenesis of neuropathic pain [87]. Further, P2Y12Rs contribute to the neuronal damage in brain ischemia [88,89] and multiple sclerosis [90].

P2Y12 and P2Y13R mRNA and protein both occur in microglia in considerable amounts; accordingly, P2Y13Rs markedly potentiate the P2Y12R-mediated chemotaxis response [91]. In addition, microglia in P2Y13R-deficient mice were less ramified than in the wild-type controls, suggesting the participation of these receptors in the regulation of microglial morphology.

#### 4.2.2. P2Y6 and P2Y4 Receptors

Whereas P2Y12 is an ADP-sensitive receptor, the agonist rank order at P2Y6Rs, the nucleotide with the highest agonist potency is UDP (UDP > UTP > ADP), and at P2Y4Rs, UTP (UTP > ATP = UDP; human) or UTP = ATP (rat, mouse) [92]. It was reported that binding of the endogenous agonist UDP to metabotropic P2Y6Rs triggers microglial phagocytosis [93]. P2Y6Rs of microglia are upregulated when neurons become damaged and send diffusible UDP signals to microglia to initiate phagocytosis and the clearing of neuronal debris. It is interesting to mention that during metabolic damage caused by mesial temporal lobe epilepsy, large concentrations of ATP are released, which may blind microglia to the ATP microgradients released by apoptotic cells as ‘find me’ signals; this causes a decreased phagocytic activity of microglia [94] (see also Section 5). Hence, UDP and ATP signalling may affect phagocytosis in opposite manners.

Pinocytosis, the internalization of fluid-phase materials, is regulated by P2Y4 rather than P2Y6Rs [95]. Another function of UDP via P2Y6R stimulation is to block the ATP-dependent migration of microglia, most likely by a shift from its migratory phenotype to a phagocytic one [96].

#### 4.2.3. P2X4 Receptors

*P2X4* and *P2X7* genes in humans are located on chromosome 12 in close proximity, indicating a tight relationship [97]. The overlapping expression of the receptor proteins has been documented in peripheral macrophages and microglia [98]. The reason for this co-expression may be the involvement of both receptors in inflammatory processes [99]. The binding affinities largely differ between P2X4 and P2X7Rs; while the former one is activated by ATP in the micromolar range, the latter one responds to ATP in the millimolar range [100]. The two receptors do not form a heteromeric receptor but appear to mutually influence each other on the molecular level and may become activated over a wide range of ATP concentrations as a P2X4-P2X7R multiprotein complex [101].

In addition to their discrimination by selective pharmacological ligands, the two related receptors exhibit at least threefold functional difference: (1) in neuronal tissue, P2X4Rs can be found in astrocytes and microglia as well as in neurons; by contrast, P2X7Rs appear to be localized in all types of glial cells, but not in neurons [102]; (2) unlike P2X7Rs, P2X4Rs are predominantly located intracellularly in lysosomal compartments of microglia [103], from where they traffic to the cell membrane under the influence of inflammatory stimuli [104]; and (3) both P2X7 and P2X4Rs form on long-lasting-activation large cytolytic pores, allowing the transmembrane passage of molecules larger than the diameter of their intrinsic channel proteins [105]. However, as opposed to P2X7Rs, the pores formed by P2X4Rs fail to induce cytoskeletal rearrangements and do not lead to cell death [105].

P2X4Rs may increase both migration and secretory properties of microglia. It was found that this receptor interacts with the P2Y12R in regulating chemotaxis; the downregulation or pharmacological blockade of P2X4Rs protracted microglial site-directed migration [83]. Spinal microglia may secrete on activation brain-derived neurotrophic factor (BDNF), causing an altered transmembrane gradient of Cl^−^ in a subpopulation of dorsal horn lamina I neurons, presumably through the downregulation of the neuronal chloride transporter KCC2 [106,107]. This in turn reverses in these neurons the polarity of GABA and glycine effects to depolarization instead of hyperpolarization, resulting in an excitability increase. Such an excitability increase manifests itself on the systemic level as neuropathic pain, with the cardinal symptoms of spontaneous pain, hyperalgesia, and tactile allodynia. Although these early investigations suffered under the low selectivity of the antagonist trinitrophenyl-ATP (TNP-ATP) for P2X4Rs versus P2X1 and P2X3Rs, the simultaneous use of antisense oligodeoxynucleotides and P2X4R-deleted mice strengthened the conclusions. It is noteworthy that, in the meantime, we have several highly selective P2X4R antagonists/negative allosteric modulators at our disposal [108].

Mechanical injury of the L4 spinal nerve induces the production of the chemokine CCL21 and its transportation via the dorsal root ganglion to the spinal cord dorsal horn, where it increases the membrane expression of intracellular P2X4Rs [109]. The extracellular matrix protein fibronectin also stimulates the upregulation of P2X4Rs in microglial cells [110]. Fibronectin acts through the activation of Lyn, a member of the Src tyrosine kinase family [111].

Microglia have been shown to shape the function of oligodendrocyte precursor cells (OPCs), the brain cells which differentiate into myelin-forming cells [112]. In consequence, microglia participated both in myelin injury and remyelination during multiple sclerosis, depending on the release of the lipid components of extracellular vesicles released from microglial cell surfaces (see also Section 4.2.4). Further, blockade of P2X4R signalling exacerbated clinical signs in the experimental autoimmune encephalomyelitis (EAE) model of multiple sclerosis; this blockade also favored microglia activation to a pro-inflammatory phenotype and inhibited myelin phagocytosis [113]. Conversely, potentiation of P2X4R signalling by the positive allosteric modulator ivermectin favored a switch in microglia to an anti-inflammatory phenotype and promoted remyelination.

In a model of kainate-induced status epilepticus (SE), SE was associated with an induction of P2X4R expression in the hippocampus, mostly localized in activated microglial cells [114]. In P2X4R-deficient mice, behavioral responses to kainate-induced SE were unaltered, but some specific features of microglial activation (cell recruitment, upregulation of voltage-dependent K^+^ channels) were impaired. Therefore, the CA1 area of the hippocampus was protected from SE-induced neuronal death in the P2X4R knockout (KO) animals when compared with their wild-type controls.

#### 4.2.4. P2X7 Receptors

The expression of P2X7R mRNA and protein in the brain is highest in microglia, with much lower quantities in astrocytes [115]. Because of its well-known low sensitivity to ATP, this receptor becomes activated only by high concentrations of ATP released/outpouring from CNS cells under pathological conditions. It was suggested about twenty years ago that some P2XR channels (P2X2, P2X4, P2X7) exhibit progressive dilation during repetitive or long-lasting stimulation by ATP and that the generated pore is permeable to high molecular weight cationic dyes [116,117]. However, it was later shown that this interpretation of the experimental data obtained by reversal potential measurements is probably misleading [101,118], especially because patch-clamp recordings failed to document a change in single-channel current amplitudes or permeation characteristics during continuous ATP application [119]. Nonetheless, convincing experimental evidence proves the opening of a gateway for the entry of large molecules (e.g., Yo-Pro) into the cell interior after P2X7R stimulation, probably via the associated protein pannexin-1 forming a hemichannel [120].

The P2X7R pore is believed to be involved in the release of neuroinflammatory cytokines. Activation of microglia stimulates the release of IL-1β in a two-step process: the first being the occupation of TLR4 by LPS, leading to accumulation of cytoplasmic pro-IL-1β, and the second one being the ATP-dependent stimulation of P2X7Rs, promoting nucleotide-binding, leucine-rich repeat, pyrin domain containing 3 (NLRP3), inflammasome-mediated caspase-1 activation, and eventually secretion of IL-1β [121]. Caspase-1 generates IL-1β from pro-IL-1β by enzymatic degradation. It is important to note that the decrease of intracellular K^+^ is a major stimulus for P2X7R-dependent NLRP3 inflammasome activation [122]. Although the maturation and release of IL-1β and IL-18 are initiated by the co-stimulation of TLR4 and P2X7Rs, activated microglia may secrete also further pro-inflammatory cytokines, such as IL-6 and TNFα by other mechanisms [123]. It is important to note that the loss of intracellular K^+^ through the P2X7Rs themselves [52,116], or via two-pore domain K^+^ channels [124], is a major stimulus for P2X7R-dependent NLRP3 inflammasome activation.

IL-1β is released from microglia, packed in extracellular vesicles of variable shape/size generated by the outward blebbing of the microglial plasma membrane [125,126]. Several lines of evidence indicate that P2X7R activation by ATP is the initiating factor of blebbing, which is furthermore dependent upon p38 mitogen-activated protein kinase; it also requires rho-associated protein kinase (ROCK) activation, causing disassembly of the cytoskeletal elements, and is associated with the P2X7R C-terminus [127].

It was reported for a mouse embryonic spinal cord preparation that the ability of microglia to proliferate depends on the presence of wild-type P2X7Rs; microglia prepared from P2X7R-deficient mice showed only moderate proliferation [128]. This suggests that the physiological proliferation of microglia observed at day 13.5 in the spinal cord motoneuron area, when microglia phagocytize dying motoneurons, is regulated by P2X7Rs [128,129]. In enriched microglial cultures, only the pore-forming variant of P2X7R was able to promote proliferation [130,131]. In the case of a point mutation in the *N*-terminus of this receptor (P2X7R-G345Y), the pore-forming and proliferation-inducing capacities were lost, although the channel function remained intact. It was also reported that the ability of P2X7Rs to drive microglial proliferation appears to depend on the release of IL-1β [130].

Microglial phagocytosis is executed in the first line by P2Y6Rs, but P2X7Rs are also involved in this cellular reaction. However, ATP inhibited rather than fostered microglial phagocytic activity in rat primary microglial cultures through the stimulation of P2X7Rs [132]. The knockdown or pharmacological blockade of P2X7Rs restored phagocytosis by ATP-treated microglia. In apparent contradiction to this finding, neural progenitor cells and neuroblasts of human fetal telencephalon could clear apoptotic cells by innate phagocytosis mediated by their P2X7Rs [133]. It was astonishing that this scavenger activity was not prevented/reversed by pharmacological P2X7R antagonists [134]. The involvement of authentic P2X7Rs in the phagocytic process was suggested among others by its typical low sensitivity to ATP and by molecular biology methods.

The loss of important cell ingredients through the P2X7R pore and the release of pro-inflammatory cytokines and chemokines with their subsequent deleterious effects may cause necrotic death of both the microglia itself (‘suicide receptor’; [135,136]), and the targeted CNS neurons by the accompanying neuroinflammation evolving during neurodegenerative illnesses (AD, [137]; PD, [138]). In addition, P2X7Rs are involved in apoptotic reactions via the activation of the caspase cascade [139]. However, the P2X7R-mediated release of TNFα (causing not only apoptosis but also proliferation) may cause neuroprotection and in consequence favors neuroregeneration, observed during long-lasting neurodegenerative illnesses [140,141].

There is a high number of recent review articles available on the involvement of microglia [8,142] and especially their P2X7Rs in neurodegenerative diseases [143,144,145]. The reader is requested to consult these reviews instead of expecting a detailed discussion of such effects in our present overview. It should be mentioned, however, that irrespective of the pathophysiology of the specific neurodegenerative disease, there is always a superimposed neuroinflammatory component that heavily depends on the overstimulation of microglial P2X7Rs. This explains the demonstrated beneficial effect of P2X7R antagonists in various in vivo and in vitro experimental models of neurodegenerative illnesses such as AD [146,147], PD [148,149], amyotrophic lateral sclerosis [150,151], multiple sclerosis [152,153], epilepsy [154,155], and ischemia [156].

#### 4.2.5. A2A and A2B Receptors

As pointed out repeatedly, microglia survey brain tissue by motile cell processes. The movement of these processes is guided by the local release of the chemoattractant ATP/ADP acting via P2Y12R stimulation. The ADP-driven process extension was reversed to process retraction during inflammation by A2A adenosine receptor upregulation coincident with P2Y12R downregulation [157]. Process retraction is indicative of the shaping of resting to amoeboid microglia and the development of a neuroinflammatory state characterized by microglial phagocytosis and the release of bioactive molecules. It was proposed that the enzymatic degradation of the originally released ATP to adenosine is causally involved in this process.

A2ARs have multiple effects in the CNS: (1) they increase the release of the excytotoxic glutamate from the respective nerve terminals, and (2) they increase the activation state of microglial cells [158]. Pharmacological blockade of A2ARs has emerged as an efficient therapeutic manipulation in various animal models of neurodegenerative disorders [146]. However, the agonistic stimulation of A2ARs may under certain circumstances also be beneficial [159]. In the following, we discuss a few examples of the predominantly neuroprotective effects of A2AR antagonists.

A2AR antagonists are effective as an adjuvant therapy to complement dopamine D2R agonistic drugs to treat PD [160]. The drug targets are supposed to be neuronal A2ARs situated at medium spiny neurons in the striatum, giving rise to the so-called indirect striatopallidal projections [53]. At the cell bodies of these neurons, A2A and D2Rs are co-localized and interact antagonistically. Thus, blockade of A2ARs enforces the effect of the diminished D2R-induced stimulation by pathologically low dopaminergic innervation from the damaged substantia nigra pars compacta. Microglia in the midbrain/substantia nigra display a higher degree of activation in patients with PD in post mortem samples and assessed with PET imaging [161], as well as in animal models of the disease [162]. Thus, A2AR antagonists certainly act at neuronal targets but also restore the activated/amoeboid, neuroinflammatory microglia to its resting/surveying state in an MPTP (selective dopaminergic neurotoxin) model of PD [163]. The consequence of this dual effect is a stronger D2R-mediated stimulation of the medium spiny neurons and additionally less degeneration of the neuronal pathways of the extrapyramidal system.

The enhancement of neuroinflammatory reactions after perinatal brain injury was observed in rats due to A2AR activation and the consequent upregulation of M1 microglial markers (IL-β, IL-6, TNF-α); an antagonist of A2ARs was beneficial under these conditions [164]. Developmental risk factors, such as the exposure to high levels of glucocorticoids, may contribute to the pathogenesis of anxiety disorders [165]. The perinatal exposure to dexamethasone of the prefrontal cortex of male rats caused hyper-ramification and increased length of microglial processes. A2AR antagonists were able to ameliorate the microglial process alterations and the accompanying anxiety behavior. Similarly, intracerebroventricular injection of an A2AR antagonist attenuated the LPS-induced increase in the level of inflammatory cytokines such as IL-1β, confirming the potentiation of neuroinflammation by A2AR agonists [166].

A2AR antagonists have been shown to protect retinal ganglion cells from microglia-induced damage in experimental models of glaucoma and ischemic retinal diseases [167]. This was shown to be due to the reversal of microglia-mediated neuroinflammation. Eventually, A2BRs augmented the production of cytokines from microglia, irrespective of their pro-inflammatory (IL-6 [168]) or anti-inflammatory nature (IL-10 [169]).

#### 4.2.6. A1 and A3 Receptors

In the CNS, A1Rs are highly expressed on microglia and as opposed to microglial A2ARs, do not induce cytoskeletal rearrangements [170,171]. In mouse microglia kept in primary culture, ATP caused, via the activation of P2X4 and P2X7Rs, a rounder cellular shape with ruffled borders; selective A1R agonists inhibited the morphological activation of microglia, probably by depressing the ATP-induced Ca^2+^ entry into these cells [171]. Similarly, the presence of A1Rs appeared to depress microglial responses to experimental traumatic brain injury (TBI; [172]) and EAE, a model of multiple sclerosis [173]. Evidence for this assumption was supplied by comparing the microglial response and neuronal damage to TBI, as well as the extent of the pro-inflammatory reactions accompanying EAE (microglial activation, demyelination, axonal injury, inflammatory gene expression) in the wild-type and KO animals. Agonists and antagonists for A1Rs confirmed the assumption that the stimulation of these receptors restricts activation of microglia during CNS injury or disease.

Microglial P2Y12Rs and A3Rs ensure a synergistic interaction between ATP and adenosine on process extension and migratory properties of this cell type [83]. Further, A3Rs attenuate neuropathic pain by suppressing the activation of microglia in the spinal cord dorsal horn [174].

#### 4.2.7. NTPDase1 (CD39) and Ecto-5′-Nucleotidase (CD73)

Microglia have been shown to release ATP, which, either by itself or after degradation to adenosine, acts on microglial receptors in a feed-back manner [175,176]. The release machinery was attributed to lysosomes endowed with a vesicular nucleotide transporter (VNUT) which transports cytosolic ATP using the proton-mediated membrane potential as a driving force [176]. Local microinjection of the relatively stable non-selective P2R agonist ATP-γ-S into a rat microglial culture caused the release of ATP through lysosomal exocytosis [175]. It was suggested that this may provide a positive feedback mechanism to generate a long-range extracellular signal for attracting distant microglia to migrate towards and accumulate at the site of ATP release being a marker for local injury.

We mentioned earlier that ATP is degraded to AMP (mainly NTPDase1) and then to adenosine (5′-nucleotidase) by a cascade of enzymes (see Section 4.1). Three related family members of NTPDase are expressed in mammalian brain [177]. NPTDase1 (CD39) hydrolyses nucleoside 5′-triphosphates and -diphosphates equally well, NTPDase3 has a threefold preference to nucleoside 5′-triphosphates over -diphosphates, and NTPDase2 hydrolyses nucleoside 5′-diphosphates only to a marginal extent. NTPDase1 has been identified as a prominent ecto-nuleotidase in the nervous tissue and had specific expression in microglia and vascular tissue [178]. It has been reported that microglial cultures prepared from CD39 KO mice exhibited low migration rates towards ATP because there was no major degradation of this ATP to ADP and eventually to adenosine on the microglial plasma membrane [179]. In other words, the ADP-sensitive P2Y12R appeared to develop its full chemoattractant activity only under co-stimulation with adenosine A3Rs [79]. Similarly, microglia in acute brain slices of CD39 KO mice had increased constitutive phagocytic activity which could not be enhanced by ATP in contrast to control animals [180]. Whereas CD39 may block the enzymatic degradation of UTP to UDP, which is an agonist to the phagocytosis regulating P2Y6Rs, it is of no surprise that ATP had no effect in this system [79].

Constitutive deletion of CD39 and CD73 or both (lower endogenous ADP and adenosine concentrations) caused an inhibition of the microglial ramified phenotype in the mouse brain with a reduction in the length of microglial processes, branching frequency, and number of intersections [181]. By contrast, the elevation of extracellular adenosine levels by inhibition of adenosine uptake by dipyridamole, the application of exogenous adenosine, and the degradation of ATP/ADP by injected apyrase, facilitated the transformation of CD39^−/−^ and CD73^−/−^ microglia into a ramified process-bearing phenotype. In conclusion, the modification of the agonist concentrations of ADP (P2Y12) and adenosine (A2A, A1/A3) receptors in the biophase around microglia by the ATP/adenosine-degrading enzymes CD39/CD73 regulated the neuroinflammation-relevant microglial functions.

CD73-derived A2AR signalling appeared to modulate microglial immunoresponses and morphological dynamics in mice [80]. The genetic inactivation of CD73 attenuated LPS-induced pro-inflammatory responses in microglia, but enhanced microglia process extension, movement, and morphological transformation in the laser injury and acute MPTP-induced Parkinson’s disease models. These findings unequivocally support the notion that A2AR activation during Parkinson’s disease may contribute to the deleterious dysbalance of A2A-D2Rs at the medium spiny output neurons in the striatum (see Section 4.2.5).

## 5. Interactions Between Microglia, Astrocytes, and Neurons via ATP/ADP and Adenosine

As mentioned previously, the extension of the ‘tripartite synapse’ hypothesis to the so-called ‘quadpartite synapse’ hypothesis states a tight interaction between neurons, astrocytes, and microglia with each other by means of neuro-/gliotransmitters and microglial-signalling molecules [37] (see Section 2.3). In the following, we enumerate further arguments supporting this interaction primarily by ATP.

In an ex vivo retinal explant system, morphological parameters and process motility of microglia were increased by ionotropic glutamatergic transmission and were decreased by ionotropic GABAergic transmission [182]. Neither glutamate nor GABA caused direct current responses on microglia, suggesting that the effects are indirect, probably via the release of ATP. In fact, the pannexin channel blocker probenecid and the wide-spectrum P2R antagonist suramin both inhibited the effects of the glutamatergic or GABAergic agonists on microglial morphology and process extension/retrieval.

In support of this idea, in hippocampal slices of mice, the application of *N*-Methyl-d-Aspartate (NMDA) triggered transient microglial process outgrowth which was blocked by the P2R antagonist reactive blue 2 and the pannexin channel inhibitor probenecid [183]. Nonetheless probenecid did not act via the blockade of pannexin channels, because a more selective inhibitor, carbenoxolone, was without effect, and genetic deletion of pannexin channels also failed to alter the NMDA-induced microglial process propulsion. The involvement of P2Y12Rs in this process was confirmed by the use of P2Y12R^−/−^ mice [184]. It was also reported that a widespread ATP release during massive neuronal hyperactivity in a mouse model of mesial temporal lobe epilepsy resulted in a ‘blinding’ of microglia, otherwise reacting to ATP gradients by the phagocytotic clearance of apoptotic neural progenitor cells in the hippocampal subgranular zone [94]. ATP release from LPS-challenged microglia differentially modulated synaptic transmission and short-term plasticity at dentate gyrus-CA3 synapses in hippocampal slices by acting on presynaptic P2X4Rs or after degradation of ATP to adenosine, on A1Rs [185].

There are also further examples of a microglia–neuron interaction by the mediation of astrocytes. Repetitive action potentials in individual layer 2/3 pyramidal neurons elicited swelling of axons, but not dendrites, which was accompanied by a large sustained depolarization of the membrane potential [186]. Microglial processes extended to these swollen axons in a mechanism involving ATP release. This extension was followed by microglial wrapping of axons that induced a soma repolarization causing neuroprotection. In another series of experiments, LPS-activated microglia released ATP, which stimulated astrocytes to release glutamate, modulating neuronal activity through the occupation of metabotropic glutamate receptors [2,187]. This was recorded as an increase in the frequency of miniature excitatory postsynaptic currents in CA1 pyramidal neurons of hippocampal brain slices. Further, when exposed to the neurotoxic methylmercury, microglia exocytosed ATP. This ATP stimulated astrocytic P2Y1Rs releasing IL-6, protecting neurons against methylmercury [188]. In fact, a bidirectional astrocyte-microglia cross-talk via ATP and inflammatory cytokines was repeatedly demonstrated [189].

Aberrant astrocyte signalling to neurons plays an important role in producing network hyperexcitability as a cause of epilepsy [190]. TNFα, possibly of microglial origin, may trigger glutamate release from astrocytes, which drives abnormal synaptic activity in the hippocampus. This chain of events was dependent on the autocrine activation of P2Y1Rs by ATP co-released with glutamate from astrocytes.

## 6. Conclusions

Microglia are definitely more than the ‘garbage man’ of the CNS, phagocyting pathogenic microorganisms and cellular debris [191]. They have important physiological and pathophysiological functions serving the homeostasis of the CNS parenchyma and the intactness of the blood–brain barrier. In contrast to primary microglial cultures, microglial cells do not appear to possess diversified receptors for all types of neurotransmitters but are instead endowed with a large array of purinergic receptors of the P2X, P2Y, and P1-classes. ATP released from nerve terminals and cell somata by vesicular mechanisms are sequentially degraded at first to ADP, then to the bioactive adenosine, and eventually to the only slightly active inosine, by an enzymatic cascade. In addition to the neurotransmitter ATP, various classic neurotransmitters such as glutamate and GABA have been shown to release ATP from astrocytes, which then may stimulate microglial cells. Thereby, a neuron/astrocyte/microglia crosstalk is operational with the participation of purinergic neuro/gliotransmitters and their respective receptors.

Microglia have important functions in neuroinflammation accompanying neurodegenerative illnesses (AD, PD, multiple sclerosis, amyotrophic lateral sclerosis, neuropathic pain, epilepsy, and ischemia). In all these illnesses, the core pathogenetic mechanism is different, but the simultaneously occurring chronic neuroinflammation is a superimposed exacerbating factor. Especially, P2X4, P2X7, and A2AR antagonists may be valuable tools to improve these conditions. However, it is noteworthy that whereas the M1 phenotype of microglia is typically releasing a host of pro-inflammatory mediators, the M2 phenotype clears cellular debris through phagocytosis and releases numerous protective factors. Whereas M1 microglia are supposed to participate in immunological defense mechanisms of the CNS, M2 microglia are involved in regenerative mechanisms going along with long-lasting neurodegeneration. Hence, microglia are by no way unequivocally injurious for the CNS.

In summary, microglia as interacting partners of neurons and neuroglial cells (astrocytes, oligodendrocytes) shape the CNS reactions to preserve homeostasis.

## Abbreviations

AD	Alzheimer’s disease
BDNF	brain-derived neurotrophic factor
CD39	NTPDase-1
CD73	ecto-5′-nucleotidase
DAMPs	danger-associated molecular patterns
EGFP	enhanced green fluorescence protein
FGF	fibroblast growth factor
IL-1β	interleukin-1β
KO	knockout
LPS	lipopolysaccharide
NGF	nerve growth factor
NLRP3	nucleotide-binding, leucine-rich repeat, pyrin domain containing 3
NTPDases	ecto-nucleoside triphosphate diphosphohydrolases
OPC	oligodendrocyte precursor cells
PAMPs	pathogen-associated molecules
PD	Parkinson’s disease
PI3K	phosphatidylinositol 3′-kinase
PLC	phospholipase C
PSD-95	postsynaptic density protein 25
R	receptor
SE	status epilepticus
TLR4	toll-like receptor 4
TNF-α	TNF-α, tumor necrosis factor-α
